# Prescribing errors in children: what is the impact of a computerized physician order entry?

**DOI:** 10.1007/s00431-023-04894-5

**Published:** 2023-03-18

**Authors:** Aylin N. Satir, Miriam Pfiffner, Christoph R. Meier, Angela Caduff Good

**Affiliations:** 1 Department of Hospital Pharmacy, University Children’s Hospital Zurich, Zurich, Switzerland; 2 Department of Pharmaceutical Sciences, University of Basel, Basel, Switzerland

**Keywords:** Prescribing errors, Children, CPOE, Patient safety

## Abstract

Prescribing errors represent a safety risk for hospitalized patients, especially in pediatrics. Computerized physician order entry (CPOE) might reduce prescribing errors, although its effect has not yet been thoroughly studied on pediatric general wards. This study investigated the impact of a CPOE on prescribing errors in children on general wards at the University Children’s Hospital Zurich. We performed medication reviews on a total of 1000 patients before and after the implementation of a CPOE. The CPOE included limited clinical decision support (CDS) such as drug-drug interaction check and checks for duplicates. Prescribing errors, their type according to the PCNE classification, their severity (adapted NCC MERP index), as well as the interrater reliability (Cohen’s kappa), were analyzed. Potentially harmful errors were significantly reduced from 18 errors/100 prescriptions (95% CI: 17–20) to 11 errors/100 prescriptions (95% CI: 9–12) after CPOE implementation. A large number of errors with low potential for harm (e.g., “missing information”) was reduced after the introduction of the CPOE, and consequently, the overall severity of potential harm increased post-CPOE. Despite general error rate reduction, medication reconciliation problems (PCNE error 8), such as drugs prescribed on paper as well as electronically, significantly increased after the introduction of the CPOE. The most common pediatric prescribing errors, the dosing errors (PCNE errors 3), were not altered on a statistically significant level after the introduction of the CPOE. Interrater reliability showed moderate agreement (*Κ* = 0.48).

*  Conclusion*: Patient safety increased by reducing the rate of prescribing errors after CPOE implementation. The reason for the observed increase in medication reconciliation problems might be the hybrid system with remaining paper prescriptions for special medication. The lacking effect on dosing errors could be explained by the fact that a web application CDS covering dosing recommendations (PEDeDose) was already in use before the implementation of the CPOE. Further investigations should focus on eliminating hybrid systems, interventions to increase the usability of the CPOE, and full integration of CDS tools such as automated dose checks into the CPOE.

**Table:** 

**What is Known:**
• *Prescribing errors, especially dosing errors, are a common safety threat for pediatric inpatients.*
•*The introduction of a CPOE may reduce prescribing errors, though pediatric general wards are poorly studied.*
**What is New:**
•*To our knowledge, this is the first study on prescribing errors in pediatric general wards in Switzerland investigating the impact of a CPOE.*
•*We found that the overall error rate was significantly reduced after the implementation of the CPOE. The severity of potential harm was higher in the post-CPOE period, which implies that low-severity errors were substantially reduced after CPOE implementation. Dosing errors were not reduced, but missing information errors and drug selection errors were reduced. On the other hand, medication reconciliation problems increased.*

**Supplementary Information:**

The online version contains supplementary material available at 10.1007/s00431-023-04894-5.

## Introduction

Medication safety and the reduction of harm due to medication is an ongoing issue in health care. The WHO addresses this problem in the Third Global Patient Safety Challenge 2017, “Medication Without Harm.” [1] Pediatric patients are at particularly high risk of experiencing medication errors, notably prescribing errors [2–5]. A meta-analysis estimated a pooled rate of 17.5% of orders containing a prescribing error [6]. Neonatal intensive care units and pediatric intensive care units (PICU) have higher rates of prescribing errors [3]. A previous study by Glanzmann et al. [7] at the PICU in our hospital revealed an error rate of 14%. The situation in pediatric general wards is poorly studied [3].

Computerized physician order entry (CPOE) seems to reduce prescribing errors [8–10], and overall evidence suggests that mortality rates and preventable adverse drug events are reduced by electronic prescribing [10].

On pediatric general wards with CPOE prescribing, errors range from 14.8 to 47.0 errors/100 prescriptions, whereas paper charts show a range from 4.1 to 58.1 errors/100 prescriptions [3]. Dosing errors are common due to the great variability of weight and size among children [5, 10, 11]. In total, 2–6% of all orders for pediatric inpatients contain a dosing error [12].

In general, studies about medication errors show a great heterogeneity in definitions and methods [3, 8, 13]. To gain useful insights, clear definitions of the studied errors should be stated, validated error classifications applied, and—in addition to the prevalence of errors—severity of harm should be assessed [4, 9, 14, 15]. Chart review rather than voluntary reporting should be used as data source [3, 13], and interrater reliability should be calculated [13].

The aim of this study was to investigate the impact of the introduction of a CPOE on prescribing errors in children in pediatric general wards. We studied the prevalence, type, and severity of prescribing errors with retrospective chart review and validated our findings by calculating the interrater reliability.

## Materials and methods

### Setting and patients

This retrospective observational study was conducted at the University Children’s Hospital Zurich, which is a tertiary care center and the largest pediatric hospital in Switzerland (220 beds). Eligible for the study were children up to 18 years who were hospitalized in 3 medical or 3 surgical wards. A total of 1000 patients, 500 from each study period, were randomly selected from 1688 (2018) and 1608 (2019) eligible patients, respectively, consisting of 250 medical and 250 surgical cases each. Patients with no prescribed medication (i.e., hospitalized for surveillance reasons) were not eligible. To control for seasonal effects, periods in identical months were included in the sampling frame: October until December 2018 (pre-CPOE) and October until December 2019 (post-CPOE).

### CPOE

Until 2019 drugs were prescribed either manually on paper charts or electronically on semi-structured charts. CGM Clinical by CompuGroup Medical Inc. was used as a hospital information system. The pediatrics-specific CPOE tool by CompuGroup Medical Inc. was further developed from the preexisting CPOE tool for adults in cooperation with members of the University Children’s Hospital Zurich.

The drug master data were provided by HospINDEX (HCI Solutions Ltd.) and validated by the hospital pharmacy. Drugs from the hospital formulary were marked and provided with appropriate routes of administration so that only these routes of administration could be selected by the prescribing physician in the ordering process. In case of drug shortages, the appropriate substitute was proposed to the prescriber.

The CPOE contained some limited clinical decision support (CDS): an automated drug-drug interaction check and a duplication check based on the data of Pharmavista by HCI Solutions Ltd. [16], which was carried out every time a drug was prescribed.

In March 2019, the CPOE was implemented on all general wards. Implementation was accompanied by user education. Thereafter, most of the medication was prescribed electronically with the CPOE. There were some exceptions that could not be prescribed with the tool for technical reasons, such as patient-controlled analgesia and others, leading to hybrid drug prescription system to a small degree.

### Information on drug dosing

Prescribers at the University Children’s Hospital Zurich had access to several guidelines and databases. They were requested to follow the dosing guidelines provided by the web application CDS by PEDeDose (PEDeus Ltd.), which was described by Higi et al. [17]. These dosing recommendations are based on the national harmonized dosing guidelines by Swisspeddose [18] and contain additional information. Aside from that, internal guidelines from different specialist fields with recommendations for certain disease patterns were available for all prescribers.

### Prescriptions and medication review

A full medication review was conducted for all included patients, whereby the rater had access to all patient data. An adapted version of the medication appropriateness index [19] was used to guide the rater through the review process (Supplement 1).

All prescriptions within the first 24 h of admission to the ward were included. For patients who were hospitalized for elective surgery, prescriptions were included in the 24 h after surgery and return to the ward from PICU. Excluded medications were parenteral nutrition, lipids, any blood cell transfusions, insulin, solutions for dialysis, solutions for fluid management such as normal saline solution, dextrose 5%, dextrose 5% in normal saline solution, or acetated ringers. Electrolytes were included, even if they were added to parenteral nutrition.

### Prescribing errors

As proposed by Dean et al. [20] the definition of a prescribing error was adopted as follows: “A clinically meaningful prescribing error occurs when, as a result of a prescribing decision or prescription writing process, there is an unintentional significant (1) reduction in the probability of treatment being timely and effective or (2) increase in the risk of harm when compared with generally accepted practice.”

Prescribing errors were classified into different types of errors according to the well-established PCNE Classification V 9.1, German Version [21, 22]. As pictured in Table 1, a subset of 7 primary domains from the PCNE Classification and 20 causes were used, which best matched the prescribing errors we found in our study. In the PCNE Classification, these causes are classified not only into the area of prescribing, but also into dispensing (PCNE 5), and patient transfer (PCNE 8). Nevertheless, we analyzed only drug prescriptions and no further steps in the medication process. A prescription could have more than one error, but a cause could only be recorded once per prescription. Supplement 2 displays how dosing errors were rated.

**Table 1:** Types of prescribing errors according to PCNE Classification V 9.1 [21]

**Primary domain**	**Code**	**Cause**
Drug selection	1.1	Inappropriate drug according to guidelines/formulary
	1.2	No indication for drug
	1.3	Inappropriate combination of drugs, or drugs and herbal medications, or drugs and dietary supplements
	1.4	Inappropriate duplication of therapeutic group or active ingredient
	1.5	No or incomplete drug treatment in spite of existing indication
	1.6	Too many drugs/active ingredients prescribed for indication
Drug form	2.1	Inappropriate drug form/formulation (for this patient)
Dose selection	3.1	Drug dose too low
	3.2	Drug dose of a single active ingredient too high
	3.3	Dosage regimen not frequent enough
	3.4	Dosage regimen too frequent
	3.5	Dose timing instructions wrong, unclear or missing
Treatment duration	4.1	Duration of treatment too short
	4.2	Duration of treatment too long
Dispensing	5.1	Prescribed drug not available
	5.2	Necessary information not provided or incorrect advice provided
Patient transfer related	8	Medication reconciliation problem
Other	9.1	No or inappropriate outcome monitoring (incl. therapeutic drug monitoring)
	9.2	Other cause; specify
	9.3	No obvious cause

To increase the clinical relevance of our findings, we also included the severity of harm due to errors, even though the retrospective rating of the potential harm is likely to be more subjective than the rating of actual harm. [9, 15]. To determine the potential severity of the detected errors, we used the NCC MERP index as adapted by Forrey et al. [15, 23, 24] (see Table 2).

**Table 2:** Severity of potential harm due to error according to NCC MERP Index adapted by Forrey et al. [25]

**Categories**	**Description**
A: Capacity to cause error	Circumstances or events have the capacity to cause error
B: Does not reach patient	An error could have occurred but the error would not reach the patient (an “error of omission” does reach the patient)
C: No harm	An error could have reached the patient but would not cause patient harm
D: No harm	The error could have reached the patient and would have required monitoring to confirm that it resulted in no harm to the patient or required intervention to preclude harm
E: Temporary harm	The error may have contributed to or resulted in temporary harm to the patient and required intervention
F: Temporary harm	The error may have contributed to or resulted in temporary harm to the patient and required initial or prolonged hospitalization
H: Temporary harm	The error may have required intervention necessary to sustain life
G: Permanent harm	The error may have contributed to or resulted in permanent patient harm
I: Death	The error may have contributed to or resulted in the patient’s death

### Interrater reliability

All patients were reviewed by the first rater (AS), and a random sample of 5% of all patients was additionally reviewed by a second rater (MP). Both raters were pharmacists with several years of experience in pediatric clinical pharmacy. A procedural manual was created to ensure that both raters approached the review in a similar way and that consistent data could be captured, as suggested by Vassar et al. [25]. Both raters decided on whether or not a prescription contained an error, which PCNE category the error belonged to, and what NCC MERP severity level it might have resulted in.

### Database and statistical analysis

The study database was built with Microsoft SQL Server 2019 Master Data Services. The personal data from all included patients were automatically exported from the hospital information system into the database.

Statistical analyses were conducted with RStudio 2022.02.1 and IBM^®^ SPSS^®^ Statistics Version 27. The sample size of 1000 patients was estimated according to a previous study by Glanzmann et al. [7], with a reduction of prescribing errors from 14 to 9% based on a power of 0.8 and a one-sided test. Patient demographic data, rates of prescribing error, and severity of harm were compared by *T*-test, chi-square test, or Mann–Whitney test. Interrater reliability was calculated with Cohen’s kappa.

## Results

### Population

Pre-CPOE and post-CPOE patients did not differ in their demographic characteristics (Table 3), except for the length of stay. Post-CPOE patients stayed significantly longer on the ward (mean: 2.5 days) than pre-CPOE (mean: 2.1 days, *p* = 0.005, *t*-test).

**Table 3:** Demographic comparison before and after CPOE

**Categories**	**Pre-CPOE** ***N *= 500**	**Post-CPOE** ***N*** **= 500**	***P*-value**
Sex			0.750
Female	219 (43.8%)	214 (42.8%)	
Male	281 (56.2%)	286 (57.2%)	
Age (in categories)			0.444
Preterm	3 (0.6%)	0 (0%)	
Neonates	19 (3.8%)	11 (2.2%)	
Infants	161 (32.2%)	167 (33.4%)	
Children	225 (45.0%)	222 (44.4%)	
Adolescents	92 (18.4%)	100 (20.0%)	
Age (mean)	Years	Years	0.549
	5.86 ± 5.39	6.06 ± 5.39	
Weight	kg	kg	0.774
	23.33 ± 19.50	23.68 ± 19.02	
Height	cm	cm	0.879
	112.05 ± 37.56	112.59 ± 38.33	
Body surface	[m^2^]	[m^2^]	0.835
	0.861 ± 0.497	0.871 ± 0.480	
Diagnosis (mean)	Quantity	Quantity	0.285
	3.12 ± 2.89	3.33 ± 3.19	
Length of stay	Days	Days	0.005*
Mean	2.08 ± 2.23	2.52 ± 2.71	
Median	1	2	

*Indicates significant value

### Drug prescriptions

A total of 5022 drug prescriptions for 1000 patients were analyzed: 2299 drug prescriptions pre-CPOE and 2723 post-CPOE. Patients were prescribed more drugs post-CPOE than pre-CPOE (mean = 5.5 drugs vs. mean = 4.6 drugs, *p* < 0.001).

Handwritten prescriptions were significantly reduced by the CPOE (38.9% vs. 0.5%, *p* < 0.001).

### Number of errors overall

A total of 2485 errors for all prescriptions was found, of which 1802 errors occurred pre-CPOE and 683 errors post-CPOE. Consequently, 78.4 errors per 100 prescriptions (95% CI: 76.2–80.6) were found before the introduction of the CPOE vs. 25.1 errors per 100 prescriptions (95% CI: 23.0–27.1) after introduction of the CPOE (*p* < 0.001, *t*-test). This means that 69.2% (95% CI: 67.4–71.0) of all prescriptions pre-CPOE contained at least one error vs. only 22.8% (95% CI: 21.2–24.5) of all prescriptions post-CPOE, which implies a significant reduction of errors (*p* < 0.001). In 100 admissions, 360 errors could be found pre-CPOE vs. 137 in post-CPOE (*p* < 0.001).

**Table 4:** Types of errors overall

**Categories**	**Pre-CPOE** ***N*** **= 1802**	**Post-CPOE** ***N*** **= 683**	***P*-value**
PCNE primary domains			
1. Drug selection	102 (5.7%)	109 (16.0%)	0.450
2. Drug form	10 (0.6%)	17 (2.5%)	0.361
3. Dose selection	203 (11.3%)	203 (29.7%)	0.088
4. Treatment duration	8 (0.4%)	5 (0.7%)	0.266
5. Dispensing	1452 (80.6%)	305 (44.7%)	< 0.001*
8. Patient transfer related	4 (0.2%)	17 (2.5%)	0.010*
9. Other	23 (1.3%)	27 (4.0%)	0.975

The most frequent type of error (80.6% of all errors pre-CPOE and 44.7% of all errors post-CPOE) was in PCNE primary domain 5 (dispensing) (Table 4). More precisely, 79.5% of all errors pre-CPOE and 44.1% of all errors post-CPOE were type 5.2 errors: “Necessary information not provided or incorrect advice provided.” Examples of this type of error pre-CPOE are: “Missing or incorrect information about the route of administration,” “active ingredient missing,” (only product name prescribed) or “drug form missing.” Post-CPOE, the most frequent error 5.2 was due to the additional selection of a mode of administration, like buccal or lingual, where it was not appropriate. Most of these errors 5.2 were of minor severity (NCC MERP severity grades A–D). Some of the 5.2 errors, though were rated as “temporary harm possible,” such as analgesic prescriptions in reserve with a frequency but no maximum number of doses prescribed.

### Severity of harm due to errors

The overall severity of harm increased significantly after the introduction of the CPOE. Mean rank pre-CPOE was 1175 vs. 1423 post-CPOE (*p* = 0.000, Mann–Whitney test).

**Table 5:** Severity of harm according to adapted NCC MERP index

**Severity NCCMERP**	**Pre-CPOE** ***N*** **= 1802**	**Post-CPOE** ***N*** **= 683**	***P*-value**
Capacity to cause error (*A*)	91 (5.0%)	18 (2.6%)	< 0.001*
Does not reach patient (*B*)	291 (16.1%)	81 (11.9%)	< 0.001*
No harm (*C* + *D*)	996 (55.3%)	297 (43.5%)	< 0.001*
Temporary harm (*E* + *F* + *H*)	422 (23.4%)	284 (41.6%)	< 0.001*
Permanent harm (*G*)	2 (0.1%)	3 (0.4%)	0.826
Death (*I*)	0	0	

*Indicates significant value

Overall, the majority of the documented errors were of minor severity (NCC MERP A–D): 76.4% pre-CPOE and 58% post-CPOE (Table 5). These errors with minor severity are unlikely to result in any harm for the patient (see the “Prescribing errors” section [20]). Therefore, we decided to focus on errors with severity E–I that might have resulted in potential harm.

### Numbers of errors causing potential harm (NCC MERP E-I)

After exclusion of errors with severity A–D, we counted a remaining total of 711 errors with potential severity of harm, E–I, which were 424 errors pre-CPOE and 287 errors post-CPOE. The overall error rate of 18.4 errors/100 prescriptions (95% CI: 16.8–19.9) before CPOE was reduced to 10.5 errors/100 prescriptions (95% CI: 9.1–12.0) after introduction of the CPOE, which was a significant reduction of the error rate (*p* < 0.001). Therefore, 16.8% of all prescriptions before CPOE contained at least one error (95% CI: 15.5–18.2), whereas the error rate after CPOE was only 9.8% (95% CI: 8.6–11.1) (*p* < 0.001). The rate of errors per 100 admissions was significantly reduced from 84 errors/100 admissions pre-CPOE to 57 errors/100 admissions post-CPOE (*p* = 0.001).

### Type of errors causing potential harm (NCC MERP E-I)

As Table 6 shows, the most frequent primary domain of errors causing potential harm pre-CPOE was domain 5 (dispensing), while post-CPOE the most frequent was domain 3 (dosing errors). Type 5 errors decreased significantly from 8.7 errors/100 prescriptions to 1.9 errors/100 prescriptions (*p* < 0.001), and type 1 errors (drug selection) decreased from 3.0 to 2.0 errors/100 prescriptions (*p* = 0.031).

**Table 6:** Types of prescribing errors according to PCNE classification with NCC MERP E-I

**PCNE primary domains and causes**	**Pre-CPOE** **[errors / 100 prescriptions]**	**Post-CPOE** **[errors / 100 prescriptions]**	***p*-value**
1. Drug selection	3.00	2.01	0.031*
1.1. Inappropriate drug	0.48	0.29	0.298
1.2. No indication for drug	0.13	0.15	0.877
1.3. Inappropriate combination of drugs	0.30	0.33	0.870
1.4. Inappropriate duplication	1.48	0.44	< 0.001*
1.5. No or incomplete drug treatment	0.61	0.73	0.589
1.6. Too many different drugs	0	0.07	0.157
2. Drug form 2.1. Inappropriate drug form/formulation	0.13	0.18	0.638
3. Dose selection	5.57	5.17	0.555
3.1. Drug dose too low	1.74	2.35	0.126
3.2. Drug dose too high	2.52	1.98	0.201
3.3. Dosage regimen not frequent enough	0.61	0.29	0.102
3.4. Dosage regimen too frequent	0.61	0.26	0.063
3.5. Dose timing instructions wrong, unclear or missing	0.09	0.29	0.086
4. Treatment duration	0.35	0.19	0.226
4.1. Duration of treatment too short	0.05	0.04	0.905
4.2. Duration of treatment too long	0.30	0.15	0.248
5. Dispensing	8.70	1.91	< 0.001*
5.1. Prescribed drug not available	0	0	
5.2. “Missing information”	8.70	1.91	< 0.001*
8. Patient transfer related 8.1. Medication reconciliation problem	0.17	0.62	0.010*
9. Other	0.43	0.44	0.975
9.1. No/inappropriate monitoring	0.26	0.37	0.506
9.2. Other cause	0.17	0.07	0.321
Total	18.35	10.52	< 0.001*

*Indicates significant value

The third domain that showed a significant change from pre- to post-CPOE was PCNE 8 “patient transfer related.” These errors increased significantly from 0.2 errors/100 prescriptions to 0.6 errors/100 prescriptions (*p* = 0.010). Type 8 errors were coded when two valid prescriptions for the same patient and time were found in different media, e.g., one CPOE prescription and one prescription on a paper chart. The doubled prescriptions contained, in some cases, the same information but sometimes slightly different dosages or instructions.

There were no statistically significant differences from pre- to post-CPOE in the other primary domains. In particular, dosing errors (type 3) showed no significant change (pre-CPOE: 5.6 errors/100 prescriptions, post-CPOE: 5.2 errors/100 prescriptions, *p* = 0.555). The PCNE causes are also displayed in Table 6.

### Handwritten vs. electronically written prescriptions causing potential harm

Pre-CPOE, we compared the error rates in handwritten and electronically written prescriptions. Handwritten prescriptions showed a significantly higher rate of errors (22.6 errors/100 prescriptions) than electronically written prescriptions (15.7 errors/100 prescriptions) (*p* < 0.001). A total of 20.9% of handwritten prescriptions contained at least one error, while only 14.2% of electronically written orders contained an error.

### Interrater reliability

Cohen’s Kappa for the agreement on whether or not an error occurred in a prescription was 0.476. This implies a moderate interrater agreement [26]. The agreement on primary domains and causes showed perfect agreement with *k* = 1.000, but for severity of harm, a kappa of only 0.158 (slight agreement) was calculated.

## Discussion

After the implementation of the CPOE, patients were prescribed more drugs than before (5.5 vs. 4.6). The fact that many adjustments to a CPOE prescription, such as an adaption of the dose, resulted in a new prescription line may have influenced this finding.

The overall error rate decreased significantly after implementation of the CPOE. This finding complies with the results of a recent systematic review in pediatrics by Koeck et al. [9], indicating that CPOE reduces prescribing errors.

The rate of potentially harmful errors (NCC MERP E-I) of 18.4% (95% CI 16.8–19.9) before introduction of the CPOE was higher than the error rate of 14%. Glanzmann et al. [7] found 9 years ago in our PICU, where a semi-structured order sheet was in use. Usually, error rates in PICUs are higher than those in general wards. One explanation for the higher error rate in our study might be that the PICU had a clinical pharmacist on rounds once or twice per week for years, while the general wards were not visited by a clinical pharmacist, except for one medical ward with irregular visits once per week at maximum. Besides that, the study of Glanzmann et al. was prospective, where uncertainties about the prescription could be clarified by direct discussion with the prescriber, whereas we conducted a retrospective study.

The CPOE especially improves the quality of prescriptions, which can be seen in the largest reduction of potentially harmful errors (NCC MERP E-I) in PCNE errors 5.2 (“lacking or wrong information”) from 8.7 to 1.9 errors/100 prescriptions (*p* < 0.001). This is consistent with the findings of Jungreithmayr et al. [27], who showed that a CPOE increased the quality of the prescription documentation.

Drug selection errors (PCNE 1) and especially cause 1.4 “inappropriate duplication of therapeutic group or active ingredient” were significantly reduced post-CPOE. This could be contributed to the duplication-check CDS.

Interestingly, cause 1.3 concerning drug-drug interactions did not decrease, even though one CDS tool (Pharmavista) offered an automatic drug-drug interaction check. This could be attributed to either alert fatigue [29] or poor usability of the integrated tool [30].

Nevertheless, a new hybrid-systems-related error did occur, as medication reconciliation problems (PCNE 8) increased significantly with remaining paper prescriptions for special medication. This type of error might be prevented through the integration of all prescriptions into the CPOE and the complete elimination of prescriptions on paper.

The CPOE had no effect on dosing errors. This finding might be explained by the fact that the web application CDS covering dosing recommendations (PEDeDose) was already in use before implementation of the CPOE, and there was no automated dose check to validate the prescriptions. The lacking effect of CPOE on dosing errors was also seen by Roumeliotis et al. [28]. Dosing errors could be prevented more effectively by a fully integrated CDS that offers dosing support and automated dose check [31].

The fact that handwritten prescriptions contained more errors than electronic prescriptions is a plausible finding, as CPOE leads to standardized, legible, and complete prescriptions [32].

The errors that occurred post-CPOE were more severe than pre-CPOE. This implies that a large number of minor-severity errors like “missing information” (PNCE 5.2) and others no longer occurred after the introduction of the CPOE. It also indicates that the introduction of the CPOE was not able to reduce the severity of harm but only the rate of errors.

## Limitations

Due to the retrospective nature of our study, we had to interpret previously recorded data on patients’ history and their prescriptions. This could have led to the conclusion that an error had occurred, although there was a plausible reason for the deviation from the norm, which was however not recorded in the patient’s charts. For this reason, the retrospective nature of our study imposes a limitation not only on the rate of errors but especially on the interpretation of the severity of harm.

Furthermore, not optimal interrater reliability imposes another limitation to our study. Even though a procedure manual existed, there was only limited training and coordination between the two raters. One rater might have tried to capture errors comprehensively, whereas the other had a more pragmatic way of assessment of the prescriptions. Rater 1 reviewed all 1000 patients and therefore had more routine in the procedure, while rater 2 only reviewed 50 patients.

## Conclusion

In conclusion, our findings imply a positive impact of the CPOE on patient safety by reducing the prevalence of prescribing errors. Especially the high number of errors with low harming potential (NCC MERP A–D) was reduced, but also potentially harmful errors were significantly reduced.

Hybrid systems of CPOE and paper charts carry a risk for errors and should therefore be eliminated. Future research might focus on interventions to increase the usability of the CPOE and on the full integration of CDS tools, for example, dosing support and notably automated dose check into the CPOE.

## Supplementary Information

Below is the link to the electronic supplementary material.

Supplementary file1 Adapted MAI (DOCX 15 KB)

Supplementary file2 Rating of dosing errors (DOCX 19 KB)

## Data Availability

The datasets generated and analysed during the current study are not publicly available due to sensitivity.

## Abbreviations

CDS: Clinical decision support
CI: Confidence interval
CPOE: Computerized physician order entry
PCNE: Pharmaceutical Care Network Europe
PICU: Pediatric intensive care unit
