# Supplementary material for: Prescribing errors in children: what is the impact of a computerized physician order entry?
Source: Eur J Pediatr. 2023 Mar 18;182(6):2567–75. doi: 10.1007/s00431-023-04894-5 (PMC10257583; doi:10.1007/s00431-023-04894-5)
Supplement: Supplementary file 1 — Supplementary file1 Adapted MAI (DOCX 15 KB) [file 431_2023_4894_MOESM1_ESM.docx]

Prescribing errors in children: What is the impact of a computerized physician order entry?

European Journal of Pediatrics

Aylin N. Satir^1^, Miriam Pfiffner^1^, Christoph R. Meier^2^, Angela Caduff Good^1^

^1^ University Children’s Hospital Zurich, Department of Hospital pharmacy, Zurich, Switzerland

^2^ University of Basel, Department of Pharmaceutical Sciences, Basel, Switzerland

Corresponding author: Aylin N. Satir, [a.satir@unibas.ch](mailto:a.satir@unibas.ch)

**Supplement 1**

**Adapted Medication Appropriateness Index (MAI)**

1. Is there an indication for the drug?
2. Is the medication effective for the condition?
3. Is the dosage correct?
4. Has therapeutic drug monitoring been prescribed (if necessary)?
5. Has the dosage been adjusted for kidney/hepatic function?
6. Are there clinically significant drug-drug interactions?
7. Are there clinically significant drug-disease/condition interactions?
8. Are the directions correct and practical?
9. Is the drug form suitable for the patient and the indication?
10. Is there unnecessary duplication with other drug(s)?
11. Is the duration of therapy acceptable?
12. Is a drug missing for an indication or as a preventive drug?
