# Supplementary material for: Prescribing errors in children: what is the impact of a computerized physician order entry?
Source: Eur J Pediatr. 2023 Mar 18;182(6):2567–75. doi: 10.1007/s00431-023-04894-5 (PMC10257583; doi:10.1007/s00431-023-04894-5)
Supplement: Supplementary file 2 — Supplementary file2 Rating of dosing errors (DOCX 19 KB) [file 431_2023_4894_MOESM2_ESM.docx]

Prescribing errors in children: What is the impact of a computerized physician order entry?

European Journal of Pediatrics

Aylin N. Satir^1^, Miriam Pfiffner^1^, Christoph Meier^2^, Angela Caduff Good^1^

^1^ University Children’s Hospital Zurich, Department of Hospital Pharmacy, Zurich, Switzerland

^2^ University of Basel, Department of Pharmaceutical Sciences, Basel, Switzerland

Corresponding author: Aylin N. Satir, [a.satir@unibas.ch](mailto:a.satir@unibas.ch)

**Supplement 2**

**Rating of dosing errors**

All drug dosages were validated by using the following databases or literature:

- PEDeDose ([www.pededose.ch](http://www.pededose.ch))
- SwissPedDose ([www.swisspeddose.ch](http://www.swisspeddose.ch))
- Drug label ([www.swissmedicinfo.ch](http://www.swissmedicinfo.ch))
- Hospital internal guidelines
- Uptodate ([www.uptodate.com](http://www.uptodate.com))
- Lexicomp ([www.lexicomp.com](http://www.lexicomp.com))
- Other

A drug dosage was categorized as an error, if the prescribed dosage deviated from the literature by more than a certain percentage (see table 1) and there was no reason evident for the deviation like altered organ function, obesity, drug-drug-interactions, or other clinical reasons.

Some deviations (column “could be an error”) were only rated as errors, if there were other risk factors that the prescribed dosage could lead to patient harm (for example reduced kidney function). If there were no risk factors evident, these dosages were not categorized as errors.

| **Active ingredient** | **Limit** | **No error** | **Could be an error** | **Error** |
| --- | --- | --- | --- | --- |
| Broad  therapeutic index | Below | 90 - 111% | 80 - 90% | < 80% |
|  | Above |  | 111 - 125% | > 125% or above maximum dosage |
| Narrow  therapeutic index | Below | 95 - 105% | 90 - 95 % | < 90% |
|  | Above |  | 105 - 111% | > 111% or above maximum dosage |

Table 1: Dosage ranges adopted from PEDeus AG (1)

1. PEDeus AG. Instructions for use, PEDeDose, 2021, accessed 21.10.2022:[41 p.]. Available from: <https://www.pededose.ch/en/file/show?filename=IFU_PEDeDose_EN>.
